# Tonsillectomy in Adults over 40 Years of Age Does Not Increase the Risk of Pneumonia: A Three-Year Longitudinal Follow-Up Study

**DOI:** 10.3390/ijerph182413059

**Published:** 2021-12-10

**Authors:** Sung Joon Park, Chanyang Min, Dae Myoung Yoo, Sei Young Lee, Hyo Geun Choi

**Affiliations:** 1 Department of Otorhinolaryngology-Head and Neck Surgery, Chung-Ang University Hospital, Chung-Ang University College of Medicine, Seoul 06973, Korea; hypocratis@gmail.com (S.J.P.); syleemd@cau.ac.kr (S.Y.L.); 2 Hallym Data Science Laboratory, Hallym University College of Medicine, Anyang 14068, Korea; joicemin@naver.com (C.M.); ydm1285@naver.com (D.M.Y.); 3 Department of Otorhinolaryngology-Head and Neck Surgery, Hallym University Sacred Heart Hospital, Hallym University College of Medicine, Anyang 14068, Korea

**Keywords:** population health, adult population, tonsillectomy, pneumonia, immunity

## Abstract

To evaluate the effect of tonsillectomy on the subsequent risk of pneumonia in an adult population, a longitudinal follow-up case control study was conducted using a national health screening cohort dataset between 2003 and 2012. A total of 1005 tonsillectomy participants were 1:4 matched with 4020 control participants for age, sex, income, and region of residence. The number of pneumonia diagnoses were counted from the index date (ID) to the date after the first-year (post-ID 1y), second-year (post-ID 2y), and third-year (post-ID 3y) periods. Simple linear regression and multiple linear regression were conducted to calculate estimated values (EVs) and 95% confidence intervals for each post-ID pneumonia and compared between the two groups. Subgroup analyses were performed according to age, sex, and the number of pneumonia cases during the year prior to the ID (pre-ID 1y). In the simple linear regression model, post-ID pneumonia did not show a significant correlation with tonsillectomy (post-ID 1y: EV = 0.003; post-ID 2y: EV = 0.007; post-ID 3y: EV = 0.013; all *p* > 0.05). In the multiple regression model, post-ID pneumonia also did not show a significant correlation with tonsillectomy (post-ID 1y: EV = 0.001; post-ID 2y: EV = 0.006; post-ID 3y: EV = 0.011; all *p* > 0.05). In the subgroup analyses, tonsillectomy did not show a significant correlation with post-ID pneumonia in either the simple linear regression or multiple linear regression models (all *p* > 0.05). Tonsillectomy performed in the adult population did not show any effect in increasing the incidence of pneumonia during the first three postoperative years.

## 1. Introduction

Tonsillectomy is one of the most commonly performed operations in both children [1] and adults [2] worldwide. In children, it has been reported that the rate of tonsillectomy is 3.4 to 4.8 per 1000 people in the U.S. [3,4] and 2.58 per 1000 people in Korea [5,6]. Moreover, it was reported in 2006 that 297,000 tonsillectomies were performed in adults out of 737,000 total tonsillectomies in the U.S. [2].

The most common postoperative complications of tonsillectomy include postoperative pain, bleeding, surgical site inflammation, and infection [2,7,8,9]. Elinder et al. reported that pediatric patients who undergo tonsillectomy due to infection compared to upper airway obstruction show a significant increase in postoperative hemorrhage and significantly more days of analgesics use [7]. Lundström et al. reported that the lowest rate of patient-reported postoperative pain is observed in tonsillotomy and the highest for tonsillectomy [8]. However, these reports did not conduct analyses on the association between tonsillectomy and posttonsillectomy infection. One recent article by Jeong et al. showed that tonsillectomy and adenoidectomy are significant risk factors for the development of pneumonia in the pediatric population [9]. However, all of these previous studies were conducted in the pediatric population, and not in adult tonsillectomy.

Because tonsils are major constituents of the mucosa-associated lymphoid organs of Waldeyer’s ring [10], there have been reports advocating the protective role of tonsils in preventing the absorption and penetration of bacteria and/or viruses through the upper respiratory tract mucosa [11,12]. It has been reported that tonsillectomy and/or adenoidectomy performed in the pediatric population is associated with an increased long-term risk of respiratory and infectious diseases [13]. Similarly, in the adult population, infection has been reported to be the most common complication in the 30-day posttonsillectomy period in the U.S., followed by surgical site complications (such as fever, localized swelling, pain, or tenderness related to surgical site infections), unplanned reintubation, substantial bleeding requiring transfusion of more than four units of packed red blood cells, prolonged ventilator use, venous thromboembolism, and renal complications [2]. Among infection, pneumonia and urinary tract infections are the two most prevalent infectious conditions in this cohort.

However, there are also controversial results on the immunologic effect of tonsillectomy in both pediatric and adult populations. A recent systematic review on the effect of tonsillectomy on immunity concluded that tonsillectomy does not negatively affect the immunity of children [14]. Moreover, it has been reported that tonsillectomy has no definite effect with respect to decreasing the number of hospital visits due to upper respiratory tract infections in either children or adults [15]. Because there have only been a few reports on the immunologic outcome of tonsillectomy in the adult population, a concrete relationship between adult tonsillectomy and pneumonia has not been established.

Therefore, the aim of this study was to evaluate the effect of tonsillectomy performed in adults on the subsequent risk of pneumonia by performing a three-year longitudinal follow-up study comparing the number of pneumonia cases after the index date (ID) between participants who had received tonsillectomy (tonsillectomy group) and participants who had not received tonsillectomy (control group) using a national cohort dataset. The tonsillectomy and control groups were matched at a 1:4 ratio by adjusting for age, sex, income, and region of residence.

## 2. Materials and Methods

### 2.1. Study Population

We used the Korean National Health Insurance Service—Health Screening Cohort data for this study, and a comprehensive explanation for this cohort is provided elsewhere [16].

### 2.2. Participant Selection

**Figure 1:**
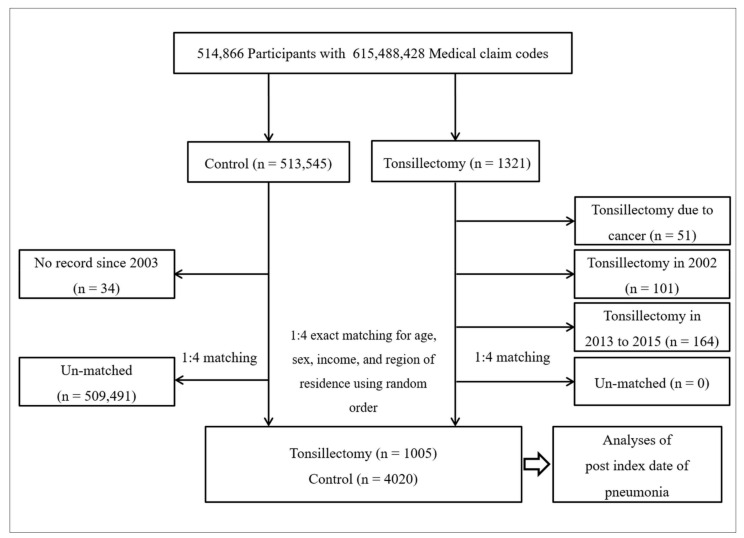
A schematic illustration of the participant selection process that was used in the present study. Of a total of 514,866 participants, 1005 tonsillectomy participants were matched with 4020 control participants for age, sex, income, region of residence, and pre-index date pneumonia for one year.

Among the 514,866 patients with 615,488,428 medical claim codes, participants were selected for the tonsillectomy group according to the definition in our study (*n* = 1321). The remaining participants were selected as a control group (*n* = 513,545). Among the tonsillectomy group, participants were excluded if the purpose of tonsillectomy was cancer (*n* = 51). To calculate the history of pneumonia from the date of tonsillectomy (i.e., the ID) to the date before a one-year period (pre-ID pneumonia for one year), participants who were treated with tonsillectomy in 2002 were also removed (*n* = 101) from the tonsillectomy group. Additionally, participants who were treated with tonsillectomy between 2013 and 2015 were excluded for the t-year follow-up period (*n* = 164). Among the control group, participants were excluded if they died before 2003 or no further record was present after 2003 (*n* = 34). The tonsillectomy participants were 1:4 matched with the control participants for age, sex, income, and region of residence. For the subgroup analyses, the tonsillectomy and control groups were additionally matched with the number of pneumonia cases diagnosed one year prior to ID (pre-ID 1y pneumonia, 0 times and ≥1 times). To minimize selection bias, the control participants were selected in a random numerical order. The ID of the control participants was set as the ID of their matched tonsillectomy participant. Therefore, each matched tonsillectomy participant and control participant had the same ID. During the matching process, 509,491 control participants were excluded. Finally, 1005 tonsillectomy participants were 1:4 matched with 4020 control participants (Figure 1).

### 2.3. Tonsillectomy (Independent Variable)

The tonsillectomy group was included if participants underwent tonsillectomy (claim code: Q2300) [17]. Because neither tonsillotomy nor partial tonsillectomy are recommended operative techniques for adult tonsillectomy in Korean Society of Otorhinolaryngology-Head and Neck Surgery, the tonsillectomy performed in this cohort was considered as conventional complete tonsillectomy utilizing various surgical techniques, including cold steel instruments, electrosurgical instruments, or low-temperature radiofrequency apparatus.

### 2.4. Pneumonia (Dependent Variable)

Pneumonia was selected based on ICD-10 codes (J12 to J18). Participants who underwent chest X-ray (claim codes: G2101-G2105, G2111, G2112, G2121, G2201, G2301-G2305, and G2322) or chest CT (claim codes: HA424, HA434, HA444, HA454, HA464, and HA474) were defined as having pneumonia. Pre-ID 1y pneumonia was categorized into 0 times and ≥1 times. The number of pneumonia diagnoses was counted from the ID to the date after the first-year period (post-ID 1y pneumonia (days 1–365)), the second-year period (post-ID 2y pneumonia (days 366–730)), and the third-year period (post-ID 3y pneumonia (days 731–1095)).

### 2.5. Covariates

The age groups were divided by five-year intervals into nine groups. The income groups were classified into five classes (class 1 (lowest income) to 5 (highest income)). The region of residence was grouped into urban and rural areas according to our previous study [18]. Tobacco smoking, alcohol consumption, and obesity represented by BMI were also categorized in the same way as in our previous study [19]. Total cholesterol (mg/dL), systolic or diastolic blood pressure (SBP or DBP; mm/Hg), and fasting blood glucose (mg/dL) were measured. The Charlson Comorbidity Index (CCI) was used with the exclusion of respiratory diseases [20,21].

### 2.6. Statistical Analyses

The general characteristics between the tonsillectomy and control groups were compared using standardized differences.

Simple and multiple linear regression were employed to calculate estimated values (EVs) and 95% confidence intervals (CIs) for post-ID 1y pneumonia, post-ID 2y pneumonia, and post-ID 3y pneumonia in the tonsillectomy group compared to the control group. Both the simple and multiple linear regressions were stratified by age, sex, income, and region of residence. In the multiple linear regression, the model was adjusted for obesity, smoking status, alcohol consumption, total cholesterol, SBP, DBP, fasting blood glucose, CCI score, asthma history, COPD history, and pre-ID 1y pneumonia.

For the subgroup analyses, we divided participants by age (<60 and ≥60 years old), sex (males and females), and pre-ID 1y pneumonia (0 and ≥1 times). Simple and multiple linear regressions were calculated.

Two-tailed analyses were performed, and significance was defined as a *p*-value less than 0.05. SAS version 9.4 (SAS Institute Inc., Cary, NC, USA) was used for statistical analyses.

## 3. Results

Compared to those of the tonsillectomy group, the age-, sex-, income-, and region of residence-matched control group did not show any significant difference in any covariates, and obesity (0.36) and CCI (0.22) showed the top two standardized differences (Table 1).

The mean number of pneumonia diagnoses one, two, and three years after the ID in both groups is shown in Table 2. In the simple linear regression model with age, sex, income, and region of residence stratification, the number of pneumonia cases diagnosed after the ID did not show a significant correlation with the presence of tonsillectomy among all of the analyzed periods (post-ID 1y: EV = 0.003, *p* = 0.812; post-ID 2y: EV = 0.007, *p* = 0.537; post-ID 3y: EV = 0.013, *p* = 0.329). In the multiple regression model with adjustment of all of the covariates and pre-ID 1y pneumonia, the number of pneumonia cases diagnosed after the index date also did not show a significant correlation with the presence of tonsillectomy within all of the analyzed periods (post-ID 1y: EV = 0.001, *p* = 0.918; post-ID 2y: EV = 0.006, *p* = 0.619; post-ID 3y: EV = 0.011, *p* = 0.408) (Table 2).

In the subgroup analyses categorized according to age, sex, and pre-ID 1y pneumonia, tonsillectomy did not show a significant correlation with the number of pneumonia cases diagnosed after the ID in either the simple linear regression model or the multiple linear regression model (all *p* > 0.05) (Table 3).

## 4. Discussion

In this longitudinal case–control study using a national cohort dataset between 2003 and 2012, the tonsillectomy group did not show a significant correlation with the incidence of pneumonia during the first three postoperative years when compared to the control group. In the subgroup analysis, the tonsillectomy group also did not show a significant association with the incidence of pneumonia during the three studied postoperative years, regardless of age, sex, or history of pre-ID 1y pneumonia.

When we searched PubMed using the term “adult tonsillectomy,” a total of 92 relevant articles written in English were searched between 1991 and 2021. Twenty-six of these 92 articles were on methods of postoperative pain control, followed by 21 articles on techniques and settings for the operation, 21 articles on the outcomes (quality of life and economic and/or social outcomes) of adult tonsillectomy, 14 articles on postoperative complications other than pain, four articles on the histopathological outcomes of adult tonsillectomy, three articles on tonsillectomy for obstructive sleep apnea (OSA), and three articles on social perceptions and tonsil volume variations. Of the 14 articles on complications other than pain, only three studies presented postoperative outcomes regarding infectious disease following tonsillectomy, and one study showed the results of children and adults altogether. Therefore, our study is the first reported outcome specifically evaluating the effect of tonsillectomy performed in adults on the postoperative incidence of pneumonia.

Several reports have shown that tonsillectomy attenuates host immunity, thereby increasing the risk of respiratory tract or infectious disease. Tonsillectomy is reported to significantly decrease the levels of serum IgA and secretory IgA in saliva [22,23,24]. Chen et al. [2] reported that pneumonia and urinary tract infection (same number of patients, 27% of complications, and 0.3% of overall participants) were the most common complications in the 30-day postoperative period among 5968 adult tonsillectomy patients. They discussed the fact that because only the adult population was included, there would be more chances of having comorbid dyspnea and respiratory disease in their cohort. Additionally, Kim et al. [5] reported, in a national cohort study, that adult tonsillectomy increased the risk of retropharyngeal and parapharyngeal abscesses (adjusted hazard ratio = 1.87, 95% CI = 1.43–2.45, *p* < 0.001) and that the tonsillectomy group showed a significantly higher cumulative probability of deep neck infection than in the control group (log rank test: *p* < 0.001). They postulated that the difference in immunologic compensation potency between children and adults resulted in an increased risk of deep neck infection in adults.

However, there have also been controversial reports on the negative effect of tonsillectomy on postoperative host immunity and respiratory or infectious disease. Kaygusuz et al. [25] reported that tonsillectomy did not compromise immune function in either the short term (three months) or long term (54 months) in a pediatric population. Additionally, Choi et al. [15] reported that tonsillectomy did not decrease the number of postoperative visits for upper respiratory infection in either children or adults. Furthermore, Chung et al. [26] showed that adult tonsillectomy significantly reduced the number of postoperative visits for acute respiratory care by 46.3% (3.4/7.3) in a Taiwanese cohort. Recently, two systematic reviews on the effect of tonsillectomy on the immune system were published. Altwairqi et al. [14] concluded that enough evidence is present to support the conclusion that tonsillectomy in the pediatric population does not have a negative effect on either humoral or cellular immunity. Moreover, Bitar et al. [27] showed more evidence suggesting that tonsillectomy does not induce negative sequalae in the immune system of either children or adults. Our study also showed that adult tonsillectomy does not have any significant effect on the incidence of postoperative pneumonia for three years. Moreover, these results were consistent regardless of age, sex, and history of pre-ID 1y pneumonia.

The probable explanation for our findings may be related to age-related changes in the immunologic function of tonsils and to the effect of tonsillectomy on the humoral immune response. Present at birth, tonsils and adenoid tissues are known to have maximal immunological activity between 4–12 years of life, and morphological and functional involution (atrophy) begins shortly after 10 years of age [28]. The age-dependent alterations of tonsillar lymphocyte subsets may reflect concomitant changes in the intensity or type of cellular or humeral immune response by tonsils. Bergler et al. [29] reported that the percentage of CD8+ T cells decreases, whereas that of CD4+ T cells increases at 2 and 65 years of age. Additionally, Lee et al. [30] showed that the rate of germinal center B cells in tonsillar B cells decreases, while the rate of memory B cells increases with aging. Moreover, they showed that the immunoglobulin isotypes in tonsils switch from IgM in children to IgA in adults. These results reflect the change in age-dependent cellular and humoral immune responses by tonsils. In addition to changes in lymphocyte subsets, Krone et al. [31] suggested, in an animal study, that alteration in the decreased innate immunity of mucosa-associated lymphoid tissue results in prolonged *Streptococcus pneumoniae* colonization caused by delayed mucosal clearance of the pathogen. With regard to the effect of tonsillectomy on the humoral immune response, van den Akker et al. [23] performed a long-term, randomized controlled trial to determine the effects of pediatric adenotonsillectomy and showed that serum immunoglobulin decreased in the immediate postoperative period, but eventually normalized. Additionally, they concluded that these changes in humoral immunity after tonsillectomy did not predispose subjects to upper respiratory infection.

Based on these findings, we can postulate that the removal of tonsil tissue in adults may result in decreased production of immunoglobulin. However, this production is rapidly recovered through compensation by other lymphoid tissues. There may be a difference in the immunologic compensation potency between children and adults. However, adult tonsils have shown decreased cytotoxic cellular immunity and germinal center reactions of humeral immunity. In addition, the impaired innate immune function of elderly tonsil tissue may only result in increased susceptibility to *S. pneumonia* infection, but not to other pathogenic organisms of pneumonia, such as *Haemophilus* or *Klebsiella*. In summary, adult tonsillectomy did not have a significant impact on host immunity and was shown to have no significant relationship with pneumonia in our study.

Rather than decreased immunity after adult tonsillectomy, the age-related decline in cell-mediated [32,33] and humoral immune responses [32,34] may have a greater impact on increasing the incidence of infectious disease. Although we did not conduct a statistical analysis, the subgroup analysis results showed that the mean number of post-ID pneumonia cases was higher in older participants during all three follow-up periods, regardless of tonsillectomy. Therefore, the involution of the immune function of tonsil tissue and the age-dependent decline in local and systemic immune responses are the potential reasons explaining the lack of a correlation between adult tonsillectomy and the incidence of pneumonia.

Several potential limitations should be considered when interpreting the present results. First, the number of pneumonia diagnoses was determined from health insurance claims, which may not have exactly reflected the number of infections. To resolve this issue, we tried to improve the reliability of pneumonia diagnosis by including only subjects who underwent chest X-ray or chest CT. Second, the etiology of tonsillectomy was not included in this study. Tonsillectomy may be performed not only due to chronic and/or recurrent tonsillitis [1], but also due to chronic tonsillar hypertrophy [5], halitosis [35], or OSA [36]. Because chronic and/or recurrent tonsillitis is of infectious etiology while the other cases are not, there is a possibility that the underlying etiology of tonsillectomy might have affected the incidence of pneumonia. Third, because the participants in our study were adults followed up from 2002 to 2015, participants who underwent tonsillectomy before 2002 or when they were children might have been included in the control group. However, considering that the annual incidence of tonsillectomy might be less than 5% in Korea, the chances are very low. Fourth, there is no concrete evidence to explain our results. However, the primary goal of this study was to reveal the potential impact of adult tonsillectomy on pneumonia, and previous studies reporting that tonsillectomy does not have a negative impact on immunity support the significance of our study results. Finally, our study was conducted using national cohort data from a single country and might not be generalizable to other ethnic groups. Further studies with diverse ethnicities are warranted to generalize our findings.

## 5. Conclusions

In conclusion, tonsillectomy performed in an adult population did not show any effect on the incidence of pneumonia during the first three postoperative years. These results were consistent regardless of age, sex, and history of pre-ID 1y pneumonia.

## Figures and Tables

**Table 1:** General characteristics of the participants.

Characteristics	Total Participants		
	Tonsillectomy (*n* = 1005)	Control (*n* = 4020)	Standardized Difference
Age (years old, *n*, %)			0.00
40–44	123 (12.2)	492 (12.2)	
45–49	286 (28.5)	1144 (28.5)	
50–54	284 (28.3)	1136 (28.3)	
55–59	175 (17.4)	700 (17.4)	
60–64	89 (8.9)	356 (8.9)	
65–69	37 (3.7)	148 (3.7)	
70–74	4 (0.4)	16 (0.4)	
75–79	6 (0.6)	24 (0.6)	
80–84	1 (0.1)	4 (0.1)	
Sex (*n*, %)			0.00
Male	686 (68.3)	2744 (68.3)	
Female	319 (31.7)	1276 (31.7)	
Income (*n*, %)			0.00
1 (lowest)	93 (9.3)	372 (9.3)	
2	100 (10.0)	400 (10.0)	
3	132 (13.1)	528 (13.1)	
4	216 (21.5)	864 (21.5)	
5 (highest)	464 (46.2)	1856 (46.2)	
Region of residence (*n*, %)			0.00
Urban	495 (49.3)	1980 (49.3)	
Rural	510 (50.8)	2040 (50.8)	
Total cholesterol level (mg/dL, mean, SD)	200.0 (36.9)	198.4 (36.4)	0.04
SBP (mm/Hg, mean, SD)	125.5 (15.0)	124.8 (15.6)	0.05
DBP (mm/Hg, mean, SD)	79.4 (11.1)	78.6 (10.6)	0.07
Fasting blood glucose level (mg/dL, mean, SD)	98.8 (33.0)	98.5 (30.6)	0.01
Obesity ^†^ (*n*, %)			0.36
Underweight	5 (0.5)	73 (1.8)	
Normal	230 (22.9)	1383 (34.4)	
Overweight	266 (26.5)	1139 (28.3)	
Obese I	437 (43.5)	1305 (32.5)	
Obese II	67 (6.7)	120 (3.0)	
Smoking status (*n*, %)			0.12
Nonsmoker	602 (59.9)	2422 (60.3)	
Past smoker	167 (16.6)	515 (12.8)	
Current smoker	236 (23.5)	1083 (26.9)	
Alcohol consumption (*n*, %)			0.01
<1 time a week	644 (64.1)	2558 (63.6)	
≥1 time a week	361 (35.9)	1462 (36.4)	
CCI score (score, *n*, %)			0.22
0	733 (72.9)	3178 (79.1)	
1	161 (16.0)	390 (9.7)	
2	67 (6.7)	207 (5.2)	
≥3	44 (4.4)	245 (6.1)	
Asthma (*n*, %)	215 (21.4)	639 (15.9)	0.14
COPD (*n*, %)	50 (5.0)	169 (4.2)	0.04
Pre-ID pneumonia for 1y (*n*, %)			0.00
0 times	990 (98.5)	3960 (98.5)	
≥1 times	15 (1.5)	60 (1.5)	
Post-ID pneumonia (mean, SD)			
First year-period	0.03 (0.27)	0.03 (0.34)	0.01
Second year-period	0.04 (0.37)	0.04 (0.36)	0.02
Third year-period	0.04 (0.50)	0.03 (0.36)	0.03

CCI: Charlson Comorbidity Index; COPD: chronic obstructive pulmonary disease; DBP: diastolic blood pressure; Pre-ID pneumonia for 1y: pneumonia history from the date of tonsillectomy treatment (index date) to the date before the one-year period; Post-ID pneumonia: the number of pneumonia diagnosis from the index date to the date after certain periods; SBP: systolic blood pressure; SD: standard deviation. Chi-square test, significance at *p* < 0.05. ^†^ Obesity (BMI (body mass index), kg/m^2^) was categorized as <18.5 (underweight), ≥18.5 to <23 (normal), ≥23 to <25 (overweight), ≥25 to <30 (obese I), and ≥30 (obese II).

**Table 2:** Simple and multiple linear regression models (estimated value (95% confidence intervals)) for the post-index date of pneumonia (post-ID pneumonia) periods in the tonsillectomy group compared to the control group.

Characteristics	Mean ± SD in Tonsillectomy Group	Mean ± SD in Control Group	Linear Regression of Tonsillectomy for Pneumonia			
			Simple ^†^	*p*-Value	Multiple ^†,‡^	*p*-Value
Post-ID 1y pneumonia	0.03 ± 0.27	0.03 ± 0.34	0.003 (−0.020 to 0.025)	0.812	0.001 (−0.021 to 0.024)	0.918
Post-ID 2y pneumonia	0.04 ± 0.37	0.04 ± 0.36	0.007 (−0.016 to 0.031)	0.537	0.006 (−0.018 to 0.030)	0.616
Post-ID 3y pneumonia	0.04 ± 0.50	0.03 ± 0.36	0.013 (−0.014 to 0.040)	0.329	0.011 (−0.016 to 0.039)	0.408

CCI: Charlson Comorbidity Index; COPD: chronic obstructive pulmonary disease; DBP: diastolic blood pressure; Pre-ID pneumonia for 1y, pneumonia history from the date of tonsillectomy treatment (index date) to the date before the one-year period; Post-ID pneumonia: the number of pneumonia diagnosis from the index date to the date after certain periods; SBP: systolic blood pressure; SD: standard deviation. Linear regression model, significance at *p* < 0.05. ^†^ Model stratified by age, sex, income, and region of residence. ^‡^ Model adjusted for obesity, smoking, alcohol consumption, total cholesterol, SBP, DBP, fasting blood glucose, CCI scores, asthma, COPD, and pre-ID pneumonia for 1y.

**Table 3:** Subgroup analyses of simple and multiple linear regression models (estimated value (95% confidence intervals)) for the post-index date of pneumonia (post-ID pneumonia) periods in tonsillectomy compared to the control group according to age, sex, and pre-index date of pneumonia for one year (pre-ID pneumonia for 1y).

Characteristics	Mean ± SD in Tonsillectomy Group	Mean ± SD in Control Group	Linear Regression of Tonsillectomy for Pneumonia			
			Simple ^†^	*p*-Value	Multiple ^†,‡^	*p*-Value
Age 40–59 years old (*n* = 4340)						
Post-ID 1y pneumonia	0.03 ± 0.25	0.02 ± 0.25	0.005 (−0.014 to 0.023)	0.625	0.004 (−0.015 to 0.023)	0.675
Post-ID 2y pneumonia	0.04 ± 0.33	0.02 ± 0.24	0.016 (−0.004 to 0.035)	0.110	0.016 (−0.004 to 0.035)	0.119
Post-ID 3y pneumonia	0.03 ± 0.49	0.03 ± 0.35	0.005 (−0.023 to 0.033)	0.734	0.003 (−0.025 to 0.032)	0.811
Age ≥60 years old (*n* = 685)						
Post-ID 1y pneumonia	0.07 ± 0.38	0.08 ± 0.69	−0.009 (−0.129 to 0.110)	0.881	−0.031 (−0.151 to 0.008)	0.607
Post-ID 2y pneumonia	0.08 ± 0.54	0.13 ± 0.75	−0.046 (−0.171 to 0.080)	0.475	−0.038 (−0.164 to 0.089)	0.558
Post-ID 3y pneumonia	0.12 ± 0.58	0.05 ± 0.43	0.068 (−0.018 to 0.153)	0.122	0.065 (−0.020 to 0.151)	0.135
Males (*n* = 3430)						
Post-ID 1y pneumonia	0.03 ± 0.28	0.02 ± 0.28	0.007 (−0.017 to 0.030)	0.579	0.007 (−0.017 to 0.030)	0.568
Post-ID 2y pneumonia	0.05 ± 0.39	0.03 ± 0.36	0.018 (−0.011 to 0.047)	0.233	0.021 (−0.009 to 0.050)	0.173
Post-ID 3y pneumonia	0.05 ± 0.59	0.02 ± 0.27	0.026 (−0.003 to 0.056)	0.082	0.026 (−0.004 to 0.056)	0.091
Females (*n* = 1595)						
Post-ID 1y pneumonia	0.03 ± 0.25	0.04 ± 0.45	−0.005 (−0.056 to 0.045)	0.833	−0.009 (−0.060 to 0.041)	0.713
Post-ID 2y pneumonia	0.03 ± 0.31	0.04 ± 0.34	−0.015 (−0.055 to 0.025)	0.466	−0.021 (−0.061 to 0.019)	0.307
Post-ID 3y pneumonia	0.03 ± 0.24	0.05 ± 0.50	−0.014 (−0.071 to 0.042)	0.625	−0.017 (−0.074 to 0.040)	0.554
No history of Pre-ID pneumonia for 1y (*n* = 4950)						
Post-ID 1y pneumonia	0.03 ± 0.26	0.02 ± 0.27	0.006 (−0.013 to 0.024)	0.559	0.003 (−0.015 to 0.022)	0.728
Post-ID 2y pneumonia	0.04 ± 0.37	0.03 ± 0.33	0.013 (−0.009 to 0.036)	0.253	0.010 (−0.013 to 0.033)	0.381
Post-ID 3y pneumonia	0.04 ± 0.49	0.03 ± 0.35	0.008 (−0.018 to 0.035)	0.542	0.006 (−0.021 to 0.033)	0.647
≥1 times history of Pre-ID pneumonia for 1y (*n* = 75)						
Post-ID 1y pneumonia	0.27 ± 0.80	0.45 ± 1.67	−0.183 (−1.085 to 0.719)	0.686	0.059 (−1.092 to 1.210)	0.918
Post-ID 2y pneumonia	0.00 ± 0.00	0.37 ± 1.13	−0.367 (−0.905 to 0.171)	0.178	−0.377 (−1.043 to 0.290)	0.261
Post-ID 3y pneumonia	0.47 ± 0.92	0.12 ± 0.56	0.350 (−0.029 to 0.729)	0.070	0.419 (−0.060 to 0.898)	0.085

CCI: Charlson Comorbidity Index; COPD: chronic obstructive pulmonary disease; DBP: diastolic blood pressure; Pre-ID pneumonia for 1y: pneumonia history from the date of tonsillectomy treatment (index date) to the date before the one-year period; Post-ID pneumonia: the number of pneumonia diagnosis from the index date to the date after certain periods; SBP: systolic blood pressure; SD: standard deviation. Linear regression model, significance at *p* < 0.05. ^†^ Model stratified by age, sex, income, and region of residence. ^‡^ Model adjusted for obesity, smoking, alcohol consumption, total cholesterol, SBP, DBP, fasting blood glucose, CCI scores, asthma, COPD, and pre-ID pneumonia for 1y.

## Data Availability

Restrictions apply to the availability of these data. Data were obtained from the National Health Insurance Service (NHIS) in South Korea and are available at https://nhiss.nhis.or.kr/bd/ab/bdaba000eng.do;jsessionid=HBIKkTbjoJBDcOtUiIGwwI0pTts092v9AhbuakX1TksVuZL2pBGTY3se6aiM6RMK.primrose22_servlet_engine10 (accessed on 6 January 2020). However only the investigators who are approved for data sharing from the Health Insurance Corporation in purpose of policy and academic research can purchase for the data and obtain access to the entire dataset.
